# Use of Promotional Language in Grant Applications and Grant Success

**DOI:** 10.1001/jamanetworkopen.2024.48696

**Published:** 2024-12-11

**Authors:** Huilian Sophie Qiu, Hao Peng, Henrik Barslund Fosse, Teresa K. Woodruff, Brian Uzzi

**Affiliations:** 1 Kellogg School of Management, Northwestern University, Evanston, Illinois; 2 Northwestern University Institute on Complex Systems, Evanston, Illinois; 3 Department of Data Science, City University of Hong Kong, Kowloon, Hong Kong SAR; 4 Novo Nordisk Foundation, Hellerup, Denmark; 5 Department of Obstetrics and Gynecology, Michigan State University, East Lansing; 6 Ryan Institute on Complexity, Kellogg School of Management, Northwestern University, Evanston, Illinois

## Abstract

**Importance:**

Scientific writing is critical for successfully showing the merits of innovative ideas to funding agencies, colleagues, and practitioners, and it has evolved over time, particularly in the increased use of promotional words.

**Objectives:**

To evaluate whether promotional language in biomedical grant writing is associated with receipt of funding and to assess who uses promotional language in their grant applications, after accounting for principal investigators (PIs), grants, and other confounders.

**Design, Setting, and Participants:**

This cross-sectional study uses previously collected data on 2439 funded and rejected National Institutes of Health (NIH) grant applications from 2007 to 2019 and 9096 funded and rejected Novo Nordisk Foundation (NNF) biomedical grant applications from 2015 to 2022, bibliographic data on the publications of each PI from OpenAlex, and fixed-effects regression analysis.

**Main Outcomes and Measures:**

Promotional language was measured using a validated dictionary of 139 science-specific terms. Grant application success was modeled as a binary outcome and was based on the percentage of promotional words, controlling for variables including characteristics of grants and PIs. The level of promotional words was modeled on the PI’s grant-related characteristics.

**Results:**

Of the 11 535 grants included in this study, the percentage of promotional words was positively associated with the probability of receiving funding (NNF grants: odds ratio, 1.47 [95% CI, 1.25-1.71]; NIH grants: odds ratio, 1.51 [95% CI, 1.10-2.11]). Younger PIs used more promotional language than the oldest PIs (1.2% vs 0.8%), men generally used more promotional language than women (1.0% vs 0.9%), and those requesting the highest funding amounts used more promotional language than those requesting the lowest funding amounts (1.1% vs 0.9%).

**Conclusions and Relevance:**

This study found that the percentage of promotional language used in medical science grants was positively associated with receiving funding after accounting for PI, grant, and year confounds and that younger PIs, men PIs, and PIs requesting higher amounts of funding generally used more promotional language in their grants. These can patterns inform research strategies for communicating the merits of good ideas to funding agencies and other researchers.

## Key Points

**Question:**

Is promotional language in biomedical grant writing associated with receipt of funding?

**Findings:**

In this cross-sectional study, regression estimates indicated that over the range of promotional words used in grant applications, the odds of being funded can increase by more than 50%. In addition, gender, age, and grant amount explained most of the variation in the use of promotional language.

**Meaning:**

This study suggests that there is an association between promotional language and biomedical grant funding success.

## Teaser

This cross-sectional study evaluates whether promotional language in biomedical grant writing is associated with receipt of funding and assesses who is more likely to use promotional language in their grant requests.

## Introduction

Grants drive advances in medical science and careers.^1,2^ Yet how the merits of grant proposals are communicated remains mysterious^2,3^ and grows in importance as federal funding levels decrease markedly,^4^ funding inequities continue,^5^ and the need for innovation in medical science intensifies.^6^ One approach espoused by policy analysts to address innovation and funding gaps is to better understand how the merits of good ideas are expressed through the language used in grant applications.^7^

A recent *JAMA Network Open* study of 1 million funded National Institutes of Health (NIH) grant proposals from 1985 to 2020 identified an unexpectedly sharp increase of over 1300% in the number of promotional words per 1 million words,^8^ prompting the conjecture that promotional language may be associated with positive funding decisions.^9,10^ Promotional words are terms that direct attention and positive sentiment toward the ideas that they characterize.^11^

Here, we conduct analyses based on prior work inside^8,12^ and outside medicine^13,14^ on the possible association between promotional language and funding success using new analyses and data. Most previous work on the factors associated with grant success has been hampered by a sample selection bias of data on only funded grants.^1,2,15^ By contrast, we analyze the full text of more than 11 000 funded and rejected biomedical grant proposals from 2 leading funders, the NIH and the Novo Nordisk Foundation (NNF), which is one of the world’s largest private funders of medical research. To focus our investigation, we examine whether medical grant funding and promotional language are associated using a newly validated promotional language lexicon. Second, we investigate which principal investigator (PI)–related factors and grant-related factors are associated with the level of promotional language in a grant proposal. Our data permit us to control for many confounders that are normally unavailable in grant data, including a PI’s prior grant success, prior productivity and citation impact, writing style, self-reported gender and age, and a range of other grant-related characteristics.

## Methods

Our NNF data contain 9096 medical science grant applications from 2015 to 2022. The NNF data include information on the final funding decision (yes or no), application year, funding amount requested, and deidentified personal information regarding the applicant’s self-reported age and gender, prior NNF grant applications, and prior NNF grant success. The mean (SD) funding amount applied for was kr4 332 081 (kr5 669 680) (current exchange rate: US $1 = kr6.89), and the mean (SD) grant proposal length was 2805 (898) words. A total of 1579 of 9096 grant applications (17.4%) are funded, of which 38.4% (3491 of 9096) are from women PIs. With the help of the NNF, the PIs’ names were merged with bibliographic data on a PI’s prior productivity and citations as reported in OpenAlex, a public bibliographic database covering nearly all of science. The NNF data were acquired through a cooperative agreement with the NNF, which is available to other researchers on request from the NNF. The study involved previously collected data and involved no interactions with participants. The Northwestern University institutional review board and the Novo Nordisk Foundation approved this study. This study followed the Strengthening the Reporting of Observational Studies in Epidemiology (STROBE) reporting guideline.

Our NIH sample includes 2439 biomedical applications submitted by the PI faculty at Northwestern University from 2007 to 2019. The university’s grant office independently deidentified the data, which include information on the funding decision, application year, amount of funding requested, and the main applicant’s gender, publication, and citation record. These NIH grant proposals have a mean (SD) requested award size of $2 028 468 ($3 005 291) and a mean (SD) length of 8452 (2912) words. According to the NIH, the mean NIH granting rate for R1 universities is 21.3% (11 052 of 51 883).^16^ In our data, the grant award rate was 19.7% (481 of 2439), of which 33.7% (823 of 2439) of are from women PIs. The NIH data were acquired through an internal university data request by the authors. eTable 1 in Supplement 1 describes definitions and measurements of all variables used in the analysis (see eAppendix 1 in Supplement 1).

The promotional language in each proposal was measured using the hand-curated, validated dictionary of 139 science-specific promotional words by Millar et al^12,17^ and Hyland and Jiang^18^ that were identified using a sample of all 901 717 published NIH grant applications from 1985 to 2020. The intricate manual coding process is described in detail by Millar et al^8,12^ and involves labeling a term as a promotional word based on whether the word could be replaced with a neutral synonym without changing the sentence’s information. Each candidate word was reviewed in 500 or more separate instances of its use by 2 independent reviewers (interrater agreement κ = 0.82). eTable 2 in Supplement 1 lists all 139 promotional terms (see eAppendix 2 in Supplement 1). The Table^12^ presents examples of promotional words and their neutral synonyms from NIH grants.

**Table.** Sample Sentences From Funded NIH Grants Showing Promotional Words Used in Different Contexts Relative to Sentences Using Neutral Synonyms in Place of Promotional Words^12^

Source	Example sentences	
	With promotional language	Without promotional language
NIH grant R01AG032179	“Further, a unique and key aspect of this program is the sharing of common mouse strains, reagents….”	“Further, a specific and central aspect of this program is the sharing of common mouse strains, reagents….”
NIH grant R35CA220436	“There remains an imperative need for more advanced PACT breast imaging technologies.”	“There remains a necessary need for more modern PACT breast imaging technologies.”
NIH grant R43EB027535	“The proposed methods offer a revolutionary innovation and will be a game-changer in the….”	“The proposed methods offer a different innovation and will be a game-changer in the….”

Abbreviations: NIH, National Institutes of Health; PACT, photoacoustic computed tomography.

### Statistical Analysis

In analyses, we measured the percentage of promotional words in a grant using an algorithm that counted the number of promotional words and divided that quantity by the total number of words in the grant. Statistical tests were conducted using Stata software (StataCorp LLC). In the NNF and NIH grant proposals, the mean (SD) percentage of promotional words per proposal was approximately 1% (0.4%). In a typical proposal of 5000 words, there was 1 promotional term every 100 words or 1 promotional word every 4 sentences (an average sentence in the data is 26 words). In a proposal’s beginning and ending sections, where first impressions and recency biases tend to affect human recall and engagement the most,^19^ promotional word density is approximately 1 term every 3 sentences. In the regressions, this variable has been multiplied by 100 to make it easy to equate a 1% change in the percentage of promotional words with a 1-unit change in the probability of being awarded a grant. We wrote a program to count the number of promotional words in a grant application. Grant application success was operationalized as a binary variable (1 = funded and 0 = nonfunded).

#### Test Procedures for Further Validation of the Lexicon

Millar et al^12^ used human raters to validate the lexicon of promotional language. To further validate the promotional word lexicon in our study context, we conducted 8 additional tests of the lexicon’s validity, internal consistency, and sensitivity measurement error using standard linguistic and computational methods.^20,21^

#### Lexicon Validation Tests

1. A standard construct validation technique is the multitrait, multimethod approach.^22^ According to the multitrait, multimethod approach, if promotional words are associated with conveying an idea’s potential importance and positive recall above what is expected from their neutral synonyms,^12^ they should have higher mean valance and arousal scores than their neutral synonyms. To compare the valance and arousal of promotional words and their neutral synonyms, we used the *Oxford English Dictionary* to identify 1013 neutral synonyms for the 139 promotional words in the lexicon of Millar et al^12^ and the validated Linguistic Inquiry and Word Count dictionary of the valance and arousal level of English-language terms.^23^ To avoid neutral synonyms of promotional language that would be inappropriate in science contexts, we removed nonscience synonyms. For example, a synonym found in the *Oxford English Dictionary* for the promotional word *revolutionary* is *rebellious*. However, *rebellious* would not be a synonym for *revolutionary* in a science context. Similarly, the words *hip* and *in vogue* are synonyms for *latest*, but would not be synonyms in the scientific context. To address this issue, we employed a native English speaker and a psychology graduate with 2 years of work experience in a university grant office to remove all nonscience context synonyms from our synonym set. Consistent with validation tests, our analysis confirmed that promotional terms have statistically higher mean valance and arousal scores than their neutral synonyms using a weighted average, *t* test, signed rank, and mvmean test (*P* = .002 in all tests). See eAppendix 3 in Supplement 1 for a list of synonyms.
2. To address the concern that promotional terms are context sensitive using computational methods, we replaced random samples of 5%, 10%, 15%, or 20% of the words from the lexicon. As in the multitrait, multimethod approach validation test, all synonyms of promotional words that are not synonyms in a scientific context were removed. For each occurrence of a promotional word in a proposal, we replaced it with a randomly selected term from the list of its neutral synonyms or the word itself. The analysis was confirmatory, showing that in all tests, the results of our analysis remained robust to the random removal of different-sized samples of words (all *P* < .001 in NNF proposals and *P* = .002 in NIH proposals).
3. To test that the lexicon instrument has internal consistency, we computed Cronbach α for all 139 promotional words based on their percentage frequencies in our data.^24^ Cronbach α confirmed an acceptable level of consistency (0.57 for NNF proposals and 0.63 for NIH proposals). Furthermore, more than 88% of promotional words in the lexicon had individual word percentage frequencies that statistically correlated with the total frequency of all other promotional words (*P* = .009).
4. To address concerns that our measure of the percentage of promotional words in a proposal was conflated with the variety of promotional words in a proposal, we recounted only the first occurrence of each promotional word so that each unique promotional word was counted at most once per proposal. This test confirmed that counting each promotional word once or as many times as it occurred in the grant did not change the regression results.
5. To address the measurement error concern that certain promotional words are simply invalid, we randomly removed promotional words from the lexicon in 5%, 10%, 15%, and 20% random samples. We then reran our analysis 100 times for each random sample. The analysis was confirmatory, showing that the dictionary was robust for up to a loss of 20% of the words. Across all 400 trails (100 times × 4 different samples), the results of our analysis remained robust to the random removal of different-sized samples of words (all *P* < .001 in NNF proposals and *P* = .006 in NIH proposals).

## Results

**Figure 1.**
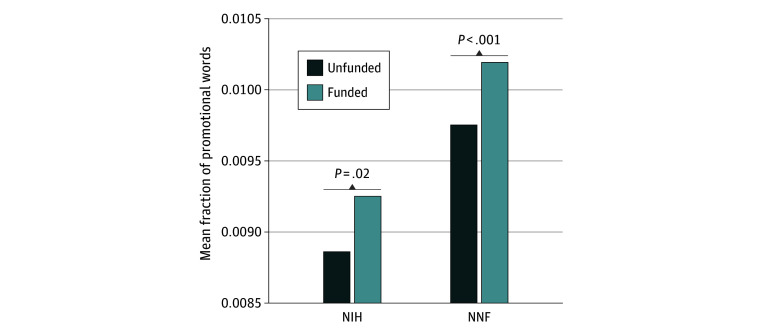
Association Between Being Awarded National Institutes of Health (NIH) or Novo Nordisk Foundation (NNF) Medical Grants and the Mean Fraction of Promotional Words in the Grant

**Figure 2.**
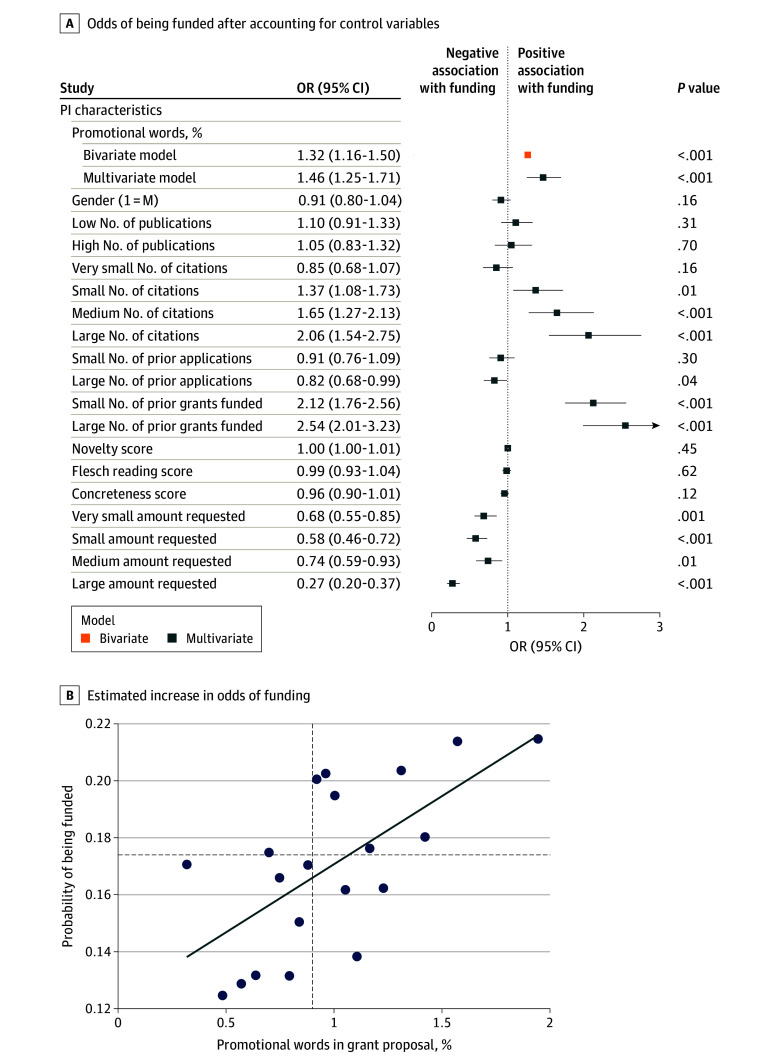
Probability of Whether a Grant Is Funded or Not Funded as a Function of the Percentage of Promotional Words in the Proposal A, Odds of being funded with the percentage of promotional words in the proposals after accounting for control variables. Odds ratios (ORs), *P* values, and 95% CIs (whiskers) are reported. Gender (1 = M) indicates that the gender variable was coded as binary, where 0 = female and 1 = male. Variables with estimated ORs that are statistically greater than 1.0 are positively associated with a grant being funded and vice versa for variables with ORs less than 1. Variables with ORs that are statistically indistinguishable from 1.0 (ie, their whiskers touch the dotted vertical line) are not associated with changes in the OR of being funded. Regression was estimated with the following fixed-effects variables: number of words and year. B, Binscatter regression showing the estimated increase in the odds of funding with changes in the percentage of promotional words in a grant. The odds of being funded can increase up to 53% for Novo Nordisk Foundation (NNF) grants. Horizontal dashed line represents the base rate for funding decisions, and vertical dashed line represents the mean percentage of promotional words in a proposal for NNF grants. Five-fold cross-validation tests confirmed that the reported results are not due to overfitting and all variance inflation factor statistics are below 10, indicating no ill-conditioning. eTable 3 in Supplement 1 presents the results for National Institutes of Health (NIH) grant proposals. Other modeling specifications of the NNF and NIH regressions are shown in eTable 2 and eTable 3 in Supplement 1, respectively, and confirm the association between promotional words and funding reported here. eTable 1 in Supplement 1 defines the variables used in the regression and reports their descriptive statistics. PI indicates principal investigator.

Figure 1 shows the raw data association between the mean percentage of promotional words in a grant and whether the grant was funded or not for NIH and NNF grant proposals. The data show that funded grant proposals contained significantly more promotional terms than did unfunded grant proposals (mean percentage of promotional words in funded vs unfunded grants: NIH, 0.93% vs 0.89%; *P* = .02; NNF, 1.02% vs 0.98%; *P* < .001), with the average grant having one promotional term every four sentences. Figure 2A and B uses multivariate logistic regression to estimate the probability of whether a grant is funded or not funded as a function of the percentage of promotional words in the grants after controlling for confounding variables, including the PI’s past grant success, productivity and citation impact, gender, grant characteristics, and year fixed effects. The odds ratios, 95% CIs, and exact *P* values are presented in the forest plot for NNF data (Figure 2A).

Promotional words were significantly associated with a positive funding decision, with an estimated odds ratio of 1.47 (95% CI, 1.25-1.71) for NNF grants and 1.51 (95% CI, 1.10-2.11) for NIH grants. In addition, we observed that the significance and magnitude of the associations are robust to control variables. We observed that the odds ratio of the percentage of promotional words did not decrease in the presence of covariates and could even increase slightly in the presence of control variables. This finding indicates that the control variables may have (1) adjusted for confounders that were distorting the association between promotional language and funding, (2) reduced residual variability, and (3) been associated with a better specified model^25^ (eTable 3 in Supplement 1).

Figure 2B plots changes in the probability of being funded as the percentage of promotional language changes. The plot uses a binscatter regression specification of the logistic model in which each scatter point represents the mean percentage of promotional words when the data are parsed into the 20 equal-sized bins. The regression line fits the scatter points.^26^ The dashed horizontal line represents the base rate of being funded for NNF grant proposals (17.4%) and the vertical dashed line represents the mean percentage of promotional words in a grant proposal for the NNF (0.9%).

Figure 2A and B indicates 3 key findings. First, among all variables in the equation, PIs’ prior academic performance and prior grant success are significant factors associated with funding and collectively explain most of the variance in the model. This finding suggests that merit and promotional words are both key factors associated with funding decisions. Second, over the range of promotional words, from the lowest to the highest value bin, the probability of being funded is estimated to increase from 14% to 22%, or a change of 53% for NNF grants. Third, grants using a below-average percentage of promotional language are funded below the base rate. Regression results for NIH grant proposals and additional NNF and NIH grant proposal regression specifications are presented in eTable 3 and eTable 4 in Supplement 1, respectively, and confirm the reported results.

**Figure 3.**
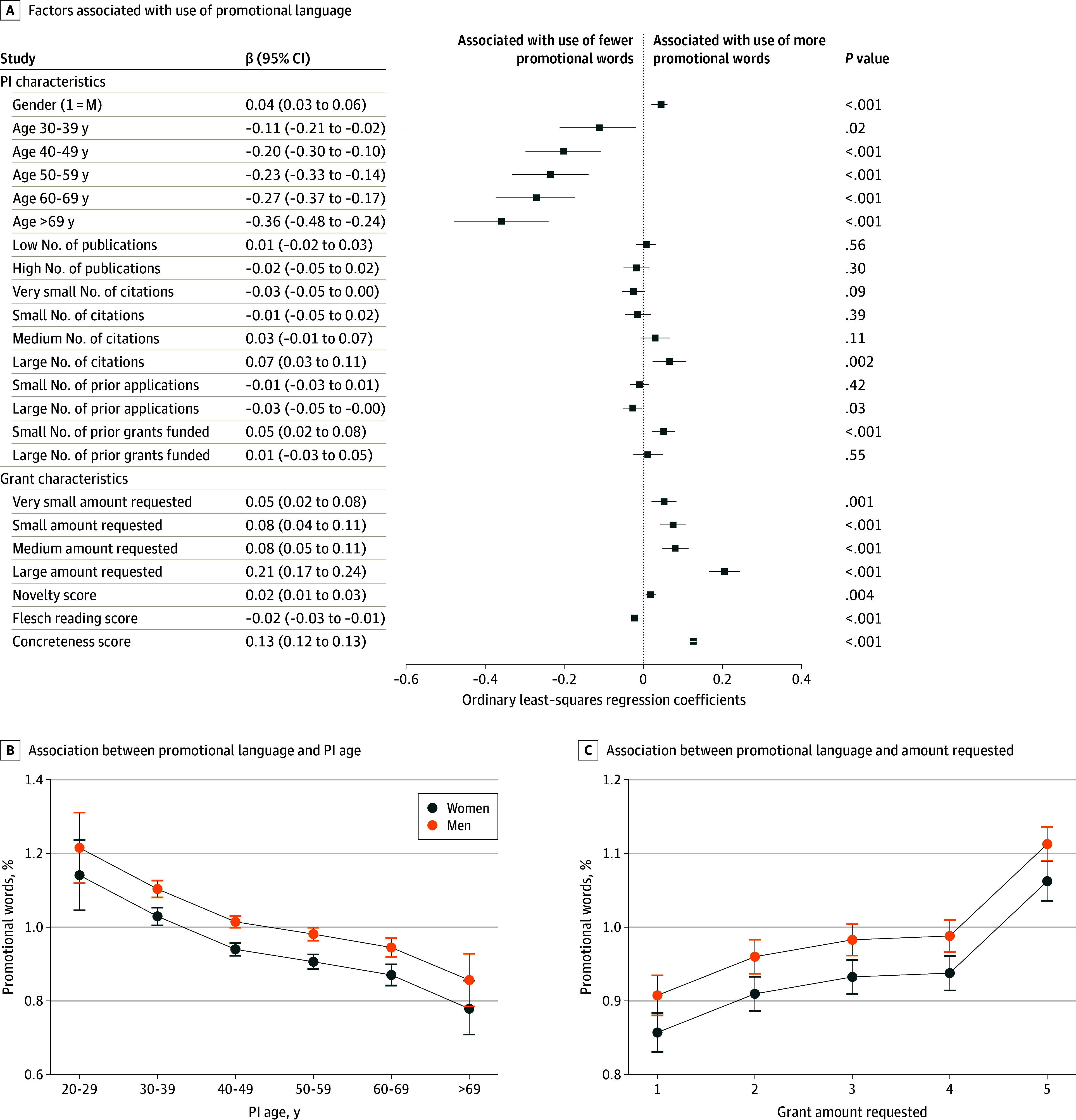
Factors Associated With the Use of Promotional Language in Grant Proposals A, Estimated factors associated with the use of promotional language in Novo Nordisk Foundation grant proposals. Gender (1 = M) indicates that the gender variable was coded as binary, where 0 = female and 1 = male. Regression was estimated with the following fixed-effects variables: number of words and year. B, Margin plot estimate of the association between the percentage of promotional language use and the PI’s age. C, Margin plot estimate of the associations between promotional language use and grant amount requested (1 indicates the lowest categorical amount requested and 5 indicates the largest categorical amount requested when the distribution of the amount of grant money requested is divided into 5 equal-size categories). National Institutes of Health results are reported in eTable 5 in Supplement 1.

To investigate the factors associated with the level of promotional word use, we used ordinary least-squares regression to regress the percentage of promotional words in a grant proposal on PI, grant, and fixed variables using the NNF data. Figure 3 presents the full regression specification and results for NNF grant proposals (see eTable 5 in Supplement 1 for NIH analysis and further details about the regression analysis). Gender, age, and grant size explain most of the variation in the use of promotional language. Figure 3B and C uses margin plots from the ordinary least-squares regression to plot the association of promotional word use with age and funding amount requested, grouped by gender.

Figure 3B indicates that age, for both women and men PIs, was inversely associated with the presence of promotional language. The youngest group of PIs was associated with an above-average use of promotional terms of nearly 20% and are associated with nearly 50% more promotional language use in their grants than the oldest group of PIs (1.2% vs 0.8%). Although men used more promotional words on average than women (1.0% vs 0.9%), the plot indicates that for the youngest group, men and women had no statistical difference in their use of promotional language, perhaps signaling that the youngest women and men scholars use promotional language equally. Figure 3C indicates that the amount of money requested was positively associated with the percentage of promotional language used in the grant: those requesting the highest funding amounts used more promotional language than those requesting the lowest funding amounts (1.1% vs 0.9%). Here, again, we observed that men used more promotional words than women at every categorical level of the funding amount requested and that the lowest- and highest-level categories showed large and significant differences while the 3 intermediate-sized grant categories are not statistically distinguishable from each other.

## Discussion

Communicating the merits of good ideas in grant applications is critical to scientific breakthroughs, careers, and graduate training, yet little is known about how language and grant funding are associated. In contrast to most science writing guidelines that discourage using adverbs and adjectives,^9^ our findings demonstrate the generalizable association between promotional language and biomedical grant funding success using more than 11 000 funded and rejected grants, controls for confounders, and data from 2 major funding agencies. After accounting for a PI’s prior publication and grant success record, promotional language can increase the odds of being funded over 50%. Although we found a statistical association between promotional language and funding, the mechanisms through which promotional language influences funding decisions require continued exploration. Another article reported that the amount of promotional language in a grant is positively associated with the grant’s inherent novelty and the citation impact of the papers based on the grant.^13^ These findings suggest that promotional language is not mainly rhetorical in nature but reflective of the grant’s innovativeness, which can improve an innovative idea’s chance of receiving funding. Nevertheless, future research should conduct causal analyses to substantiate the mechanisms behind the association between promotional language and grant success. For example, if more data become available on the review process itself, qualitative analyses of review panel dynamics and experiments could be used together to identify causal mechanisms and calibrate effect sizes. Another area of future research could focus on whether gender and age differences in promotional language use are associated with persistent gender gaps in funding and patenting equity.^1^ Last, while this study examined grants, a launching pad for many scientific studies, the differences between grants and articles and patents call for more research on promotional language to further inform medical science policies on innovation.

## Limitations

This study has some limitations. While it uses unique data on funded and unfunded grants from different funding agencies, which use different review practices and draw on different samples of researchers that broaden the representativeness of our findings across contexts, more analysis of other agency data would be useful in further confirming our findings.

## Conclusions

This survey study revealed a correlation between the percentage of promotional language in grant proposals and the likelihood of funding success, with applications featuring a higher fraction of promotional words having increased odds of being funded. Our analysis also revealed that men tend to use more promotional language than women across various funding levels. This finding underscores the significant association of linguistic style alongside scientific merit in determining funding outcomes. The results highlight the necessity for both grant writers and funders to be cognizant of how linguistic elements can influence decision-making processes in the allocation of research funds.

## Notes

Supplement 1. **eAppendix 1.** Data and Measurement **eTable 1.** Variable Definitions and Operationalizations **eAppendix 2.** Promotional Words Lexicon **eTable 2**. Lexicon of 139 Promotional Words by Eight Overarching Categories and Sample Sentences from Actual NIH Grants Showing Examples of Promotional Words Used in Different Contexts **eAppendix 3.** Promotional Words Neutral Synonyms Used in Validation Tests **eTable 3.** Logistic Regression of the Odds of Being Funded (NNF Grants) **eTable 4.** Logistic Regression of the Odds of Being Funded (NIH Grants) **eTable 5.** Predictors of the Percentage of Promotional Words in a Grant (NIH)
eReferences.

Supplement 2.
Data Sharing Statement
