# Supplementary material for: Use of Promotional Language in Grant Applications and Grant Success
Source: JAMA Netw Open. 2024 Dec 11;7(12):e2448696. doi: 10.1001/jamanetworkopen.2024.48696 (PMC11635532; doi:10.1001/jamanetworkopen.2024.48696)
Supplement: Supplement 1. — eAppendix 1. Data and Measurement eTable 1. Variable Definitions and Operationalizations eAppendix 2. Promotional Words Lexicon eTable 2. Lexicon of 139 Promotional Words by Eight Overarching Categories and Sample Sentences from Actual NIH Grants Showing Examples of Promotional Words Used in Different Contexts eAppendix 3. Promotional Words Neutral Synonyms Used in Validation Tests eTable 3. Logistic Regression of the Odds of Being Funded (NNF Grants) eTable 4. Logistic Regression of the Odds of Being Funded (NIH Grants) eTable 5. Predictors of the Percentage of Promotional Words in a Grant (NIH) eReferences. [file jamanetwopen-e2448696-s001.pdf]

Supplementary Online Content

Qiu HS, Peng H, Fosse HB, Woodruff TK, Uzzi B. Use of promotional language in grant applications and grant success. *JAMA Netw Open.* 2024;7(12):e2448696. doi:10.1001/jamanetworkopen.2024.48696

eAppendix 1. Data and Measurement
eTable 1. Variable Definitions and Operationalizations
eAppendix 2. Promotional Words Lexicon
eTable 2. Lexicon of 139 Promotional Words by Eight Overarching Categories and Sample Sentences from Actual NIH Grants Showing Examples of Promotional Words Used in Different Contexts
eAppendix 3. Promotional Words Neutral Synonyms Used in Validation Tests
eTable 3. Logistic Regression of the Odds of Being Funded (NNF Grants)
eTable 4. Logistic Regression of the Odds of Being Funded (NIH Grants)
eTable 5. Predictors of the Percentage of Promotional Words in a Grant (NIH)
eReferences.

This supplementary material has been provided by the authors to give readers additional information about their work.

© 2024 Qiu HS et al. *JAMA Network Open.*

eAppendix 1. Data and Measurement

The NNF data was shared with us in its entirety per our agreement to analyze their data for research purposes. We received no payment for the analysis from the NNF. The NIH data came from the grant office records of Northwestern University. The grant data consists of all submitted NIH grants from 2011 to 2022. The NIH data were deidentified by the grant office's internal staff. The data were analyzed statistically using mainly regression analysis and inferential statistics. All NNF and NIH data were archival and involved no interaction with subjects. The SI now contains a table (shown below) that lists all variables, their definitions and descriptive statistics. The ethics and privacy procedures for analysis were approved by Northwestern IRB (STU00219074 & STU00215754). Additionally, the NNF grant applicants all signed consent forms to have their applications analyzed by NNF or outside researchers or contractors (Website).

© 2024 Qiu HS et al. *JAMA Network Open.*

eTable 1. Variable Definitions and Operationalizations. This table describes the variables used in our analysis. Variables that have highly skewed distributions, such as “PI’s number of citations,” were operationalized as multi-category fixed effect variables as noted in the Table. The mean and standard deviation of each variable represents the distributional information of the raw data variable. The NIH data did not contain three variables that were in the NNF data as indicated in the Table.

		NNF		NIH	
Variable name	Definition and notes	Mean	S.D.	Mean	S.D.
Funded	Funding decision is coded as 1 if proposal was funded and 0 if was not funded.	17.4%	.37	20%	.37
Innovativeness Novelty Score	Calculated using a widely used and validated novelty measure that characterizes whether a grant combines past knowledge in familiar or novel ways. Information on how this measure is computed and examples of its use can found here[2-7]	9.99	11.62	n/a	n/a
%. of promotional words	Proposal’s total number of promotional words ÷ the proposal’s total words. In the regressions, this variable has been multiplied by 100 to make it easy to equate a one percent change in the percentage of promotional words with a one unit change in probability of being awarded a grant more directly interpretable.	0.0089	.43	0.009	.33%
No. of words	Proposal’s total number of words operationalized as five equal-sized categories.	2805	898	8452	2112
Concreteness score	Measure how concrete the proposal is written. This variable is computed based on the grant’s full text using the Brysbaert concreteness score [8].	-.089	1.17	-.021	.996
Flesch reading score	The grade-level needed to understand the grant’s text. This variable is computed from the grant’s full text; higher scores indicate clearer writing.	4.53	88.38	-8627	-3108
Funding amount applied for	Proposal’s amount of funding applied for operationalized as 5 equal sized fixed effect variable.	4332081	5669680	2028468	3005291
Applicant’s gender	PI’s self-reported gender.	61%		66%	
No. of prior applications	PI’s number of previous applications submitted to funding agency operationalized as 3 equal sized fixed effect variable. (NNF only)	1.7	2.5	n/a	n/a
No. of prior grant successes	PI’s number of previously funded applications submitted to funding agency operationalized as 3 equal sized categories.	.33	.84	n/a	n/a
No. of prior publications	PI’s number of publications taken from as reported in OpenAlex operationalized as 3 equal sized categories	61.9	89.35	123.1	154
No. of prior citations	PI’s number of citations taken as reported in OpenAlex bibliometric database. operationalized as 5 equal sized categories.	2650.4	6099.8	4970.11	8713.4
Application year	Year of application measured as fixed effect.	2015-2022		2011-2020	

© 2024 Qiu HS et al. *JAMA Network Open.*

eAppendix 2. Promotional Words Lexicon

Millar et al. 2022 created a dictionary of scientific promotional language by analyzing the linguistic content of all 901,717 published NIH grant applications from 1985 to 2020[9,10]. To manually code and validate the dictionary, Millar used two independent experts to evaluate each candidate promotional word based on at least 500 different instances of its contextual use and substitutability with a neutral synonym (interrater reliability was high; Cohen's k = 0.82). This process resulted in a final set of 139 scientific promotional words such as "emerging", "substantial", and "powerful". The lexicon of promotional words identified by Millar et al.[9,10] is exemplified in eTable 2 and a full list of the 139 words can be found in Millar et al.'s article online.

eTable 2. Lexicon of 139 Promotional Words by Eight Overarching Categories and Sample Sentences from Actual NIH Grants Showing Examples of Promotional Words Used in Different Contexts. Each promotional word is italicized and underscored. Lexicon and examples are from Millar et al.[10].

**Category**	**Promotional Words**
Importance	compelling, critical, crucial, essential, foundational, fundamental, imperative, important, indispensable, invaluable, key, major, paramount, pivotal, significant, strategic, timely, ultimate, urgent, vital
Novelty	creative, emerging, first, groundbreaking, innovative, latest, novel, revolutionary, unique, unparalleled, unprecedented
Rigor	accurate, advanced, careful, cohesive, detailed, nuanced, powerful, quality, reproducible, rigorous, robust, scientific, sophisticated, strong, systematic
Utility	accessible, actionable, deployable, durable, easy, effective, efficacious, efficient, generalizable, ideal, impactful, intuitive, meaningful, productive, ready, relevant, rich, safer, scalable, seamless, sustainable, synergistic, tailored, tangible, transformative, user-friendly
Quality	ambitious, collegial, dedicated, exceptional, experienced, intellectual, longstanding, motivated, premier, prestigious, promising, qualified, renowned, senior, skilled, stellar, successful, talented, vibrant
Scale	ample, biggest, broad, comprehensive, considerable, deeper, diverse, enormous, expansive, extensive, fastest, greatest, huge, immediate, immense, interdisciplinary, international, interprofessional, largest, massive, multidisciplinary, myriad, overwhelming, substantial, top, transdisciplinary, tremendous, vast
Attitude	attractive, confident, exciting, incredible, interesting, intriguing, notable, outstanding, remarkable, surprising
Problem	alarming, daunting, desperate, devastating, dire, dismal, elusive, stark, unanswered, unmet
Examples sentences with Promotional Words	"Further, a *<u>unique</u>* and *<u>key</u>* aspect of this program is the sharing of common mouse strains, reagents…"<br><br>"There remains an *<u>imperative</u>* need for more *<u>advanced</u>* PACT breast imaging technologies."<br><br>"Addressing this severe knowledge gap in one of the most *<u>fundamental</u>* aspects of cytoskeletal biology is *<u>paramount</u>* to understanding how actin functions in cells."<br><br>"The proposed methods offer a *<u>revolutionary</u>* innovation and will be a game-changer in the…"<br><br>"These *<u>innovative</u>* and *<u>novel</u>* studies will provide essential new information about the regulation of…"<br><br>"We propose to go deep in analyzing a very *unique* and *unprecedented* large scale human genomic data set for ageing research."

© 2024 Qiu HS et al. *JAMA Network Open.*

eAppendix 3. Promotional Words Neutral Synonyms Used in Validation Tests

The non-promotional synonyms used for each promotional word are:

accessible: achievable, acquirable, approachable, attainable, available, comprehensible, fathomable, graspable, intelligible, obtainable, penetrable, procurable, reachable, realizable, securable, understandable, welcoming
accurate: authentic, close, correct, error-free, errorless, exact, fact-based, factual, fair, faithful, faultless, literal, precise, precision, realistic, reliable, right, specific, sure, truthful, unambiguous, unerring, valid, veracious
actionable: corrupt, culpable, delinquent, felonious, fraudulent, illegal, illegitimate, illicit, lawbreaking, nefarious, unlawful, villainous
advanced: futuristic, higher, higher-level, modern, new, newfangled, state-of-the-art
alarming: agitating, alerting, disconcerting, disquieting, distressing, disturbing, fearing, flustering, frightening, frighting, panicking, scary, shocking, terrifying, unease, upsetting
ambitious: arduous, aspiring, avid, challenging, committed, demanding, desirous, difficult, driven, eager, enterprising, exacting, formidable, go-ahead, go-getting, hankering, hoping, hungry, impatient, itching, onerous, pioneering, progressive, purposeful, pushy, stiff, strenuous, striving, zealous
ample: abundant, adequate, capacious, commodious, copious, enough, flush, generous, plentiful, profuse, roomy, sizeable, sufficient, teeming, wide
attractive: fascinating, fetching, inviting, pleasing, prepossessing, striking, stunning
biggest: consequential, far-reaching, high-priority, hugest, megalithic, mightiest, prodigious, robustest, sizeable, solidest, sturdiest, vastest
broad: across, all-embracing, approximate, broad-ranging, coarse, conspicuous, dirty, earthy, filthy, fuzzy, general, immeasurable, improper, inclusive, indecent, indecorous, indefinite, indelicate, loose, manifest, naughty, non-specific, noticeable, obvious, overall, overt, patent, plain, prominent, pronounced, racy, ribald, risqué, rough, rude, sizeable, sweeping, unfocused, unmistakable, unrefined, unspecific, unsubtle, vague, vulgar, wide, wide-ranging
careful: attentive, cautious, conscientious, deliberate, diligent, fastidious, hypervigilant, judicious, methodical, mindful, orderly, perfectionist, prudent, scrupulous, sedulous, sensible, vigilant, wary, watchful
cohesive: adhering
compelling: cogent, conclusive, credible, engrossing, fascinating, influential, persuasive, plausible, potent, profound, reasonable, reasoned, valid
comprehensive: all-inclusive, broad-based, complete, diversified, eclectic, inclusive, indiscriminate, wide
confident: assured, hopeful, optimistic, positive
considerable: appreciable, eminent, influential, much, noted, noteworthy, prominent, sizeable
creative: accomplished, experimental, genius, gifted, imaginative, innovational, innovatory, inventive, original
critical: analytic, analytical, commentative, criticizing, dangerous, deciding, decisive, disapproving, disparaging, evaluative, expository, grave, high-priority, negative, perilous, precarious, pre-eminent, reproving, risky, uncertain, unfavourable
crucial: deciding, decisive, determining, high-priority, mandatory, necessary, needed, pre-eminent, pressing, required, requisite
daunting: disconcerting, formidable, intimidating
dedicated: allocated, assigned, committed, devoted, faithful, resolute, staunch, steadfast, unwavering
deeper: absorbed, bass, deep-rooted, deep-seated, downwards, hidden, intenser, inwards, lower, low-pitched, obscurer, opaquer, profound, recondite, richer, riveted, steeped, wholehearted
desperate: dangerous, despairing, grave, perilous, precarious, pressing, risky, wanting
detailed: all-inclusive, complete, full
devastating: calamitous, cataclysmic, catastrophic, destructive, disastrous, distressing, incisive, ruinous, shattering, shocking, striking, stunning, terrible
dire: appalling, awful, distressing, drastic, dreadful, extreme, frightful, grave, grim, harrowing, horrible, ominous, portentous, pressing, shocking, terrible
dismal: abject, awful, bad, bleak, dark, desolate, dim, dingy, dispirited, doleful, downcast, drab, dreadful, dull, forlorn, glum, grim, lamentable, melancholy, pitiful, poor, sombre, terrible, woeful, wretched
diverse: manifold, multiple, sundry, various
durable: abiding, continuing, enduring, indestructible, lasting, long-lasting, long-term, persistent, persisting, resistant
easy: facile, painless, simple, unchallenging, uncomplicated, unconstrained, undemanding, undisturbed

© 2024 Qiu HS et al. *JAMA Network Open.*

effective: closing, concluding, constructive, effectual, end, functional, implicit, implied, operative, plausible, potent, practical, tacit, valid
efficacious: constructive, effectual, functional, potent
efficient: coherent, cost-effective, energy-efficient, energy-saving, fuel-efficient, labour-saving, logical, methodical, orderly, streamlined, structured, systematized
emerging: appearing, transpiring, unfolding
enormous: wide
essential: cardinal, characteristic, chief, compulsory, consequential, elemental, high-priority, inherent, innate, intrinsic, mandatory, necessary, needed, obligatory, pre-eminent, pressing, primary, principal, quintessential, required, requisite, rudimentary, underlying
exceptional: abnormal, atypical, excellent, prodigious, rare, singular, special, uncommon, unexpected, unusual
exciting: inspiring, invigorating, moving, rousing, stimulating
expansive: all-embracing, cross-disciplinary, inclusive, sweeping, wide, wide-ranging
experienced: accomplished, adept, adroit, consummate, expert
extensive: complete, immeasurable, large-scale, profound, sizeable
fastest: quickest, rapidest, speediest, swiftest
first: basal, beginning, best, chief, earliest, elemental, firstly, foremost, foundation, highest, initial, initiatory, introductory, launching, leading, main, novelty, opening, original, primary, prime, principal, rather, rudimentary, sooner, superlative, topmost, underlying, uppermost, utmost
fundamental: basal, deciding, decisive, elemental, foundational, high-priority, pre-eminent, root, rudimentary, underlying
greatest: accomplished, adeptest, adroitest, amplest, appreciable, broadest, complete, dominant, eminent, exceedingly, extraordinarily, extremely, immensely, imposing, impressive, influential, potent, pre-eminent, proficient, prominent, pronounced, redoubtable, remarkably, salient, sizeable, strongest, tremendously
huge: prodigious
ideal: archetypal, complete, conceptual, consummate, exemplary, faultless, flawless, impracticable, model, notional, philosophical, quintessential, supreme, theoretical, unachievable, unattainable, unfeasible
immediate: close, closest, current, expeditious, instant, instantaneous, near, nearest, on-the-spot, present, primary, prompt, quick, rapid, recent, speedy, swift
immense: prodigious
imperative: commanding, exigent, necessary, peremptory, pressing
important: cardinal, chief, consequential, dominant, far-reaching, foremost, formidable, high-level, influential, main, necessary, overriding, predominant, prime, principal, salient, supreme, top-level, useful, valuable, valued
incredible: imposing, impressive, prodigious, staggering
indispensable: compulsory, consequential, high-priority, mandatory, necessary, needed, obligatory, pre-eminent, pressing, required, requisite
innovative: avant-garde, experimental, forward-looking, fresh, futuristic, groundbreaking, innovational, innovatory, inventive, modern, new, original, pioneering, progressive, state-of-the-art, trailblazing, unconventional, unorthodox, unusual
intellectual: cognitive, mental, scholarly
interdisciplinary: cross-disciplinary
interesting: engrossing, fascinating
international: global, intercontinental, worldwide
intriguing: drawing, fascinating
intuitive: instinctive, instinctual, intuitional
invaluable: irreplaceable
key: central, chief, decisive, dominant, leading, main, prime, principal, salient
largest: abundant, broadest, bulkyist, far-reaching, heftiest, hugest, large-scale, macroscale, sizeable, solidest, sufficient, tallest, vastest, wide-ranging, wide-reaching, widest
latest: contemporary, current, fresh, modern, modernistic, newest, present-day, state-of-the-art
longstanding: established, long-established
long-standing: established, long-established
major: best, capital, cardinal, chief, difficult, eminent, foremost, leading, main, pre-eminent, prime, principal, supreme, sweeping, top-tier, uppermost, utmost
massive: bulky, prodigious, staggering
meaningful: consequential, expressive, pithy, pointed, purposeful, sincere, valid, worthwhile

© 2024 Qiu HS et al. *JAMA Network Open.*

motivated: encouraged, excited, galvanized, inspired, roused, spurred, stimulated
multidisciplinary: complete, profound
myriad: countless, immeasurable, innumerable, legion, limitless, manifold, many, multifarious, multiple, multitudinous, numberless, numerous, several, sundry, unlimited, unnumbered, various
notable: acclaimed, celebrated, consequential, conspicuous, eminent, esteemed, glaring, honoured, impressive, influential, marked, memorable, noted, noteworthy, obvious, particular, pre-eminent, prominent, pronounced, rare, respected, signal, special, striking, uncommon, unusual
novel: avant-garde, different, fresh, futuristic, groundbreaking, imaginative, innovational, innovatory, inventive, modern, new, original, pioneering, state-of-the-art, trailblazing, unconventional, unorthodox, unusual
outstanding: celebrated, consequential, detectable, discernible, distinctive, due, eminent, excellent, formidable, historic, impressive, incomplete, left, memorable, neglected, noteworthy, noticeable, observable, omitted, ongoing, overdue, pending, perceivable, perceptible, pre-eminent, remaining, signal, special, striking, superlative, undischarged, undone, unfinished, unsettled, visible, vivid
overwhelming: formidable, inordinate, prodigious, profound, profuse, shattering, staggering, sweeping
paramount: incomparable, inimitable, matchless, peerless, unequalled, unexcelled, unmatched, unsurpassed
pivotal: central, deciding, decisive, determining, focal
powerful: cogent, commanding, consuming, dominant, dynamic, formidable, impressive, influential, persuasive, potent, redoubtable, solid, striking, vigorous
premier: best, chief, choice, elite, excellent, foremost, head, highest, high-grade, leading, main, peerless, pre-eminent, primary, prime, principal, quality, select, superior, superlative, top-class, top-grade, top-quality, top-ranking, top-tier, unexcelled, unsurpassed
prestigious: acclaimed, celebrated, eminent, esteemed, estimable, exalted, honoured, imposing, impressive, influential, leading, prominent, reputable, respected
productive: constructive, fertile, gainful, gratifying, helpful, high-yielding, inventive, profitable, rewarding, useful, valuable, worthwhile
promising: encouraging, favourable, hopeful, optimistic, positive, propitious, reassuring, rising, up-and-coming
qualified: bounded, cautious, certificated, certified, chartered, circumscribed, conditional, contingent, equivocal, licensed, limited, restricted, tentative
ready: achievable, acquirable, apt, arranged, attainable, available, completed, disposed, done, equipped, fast, finished, given, inclined, likely, minded, organized, predisposed, prepared, primed, procurable, prompt, prone, quick, rapid, realizable, securable, set, speedy, swift, unhesitating, willing
relevant: admissible, applicable, apposite, apropos, germane, pertinent
remarkable: prodigious, stunning
renowned: acclaimed, celebrated, eminent, esteemed, pre-eminent, prominent
revolutionary: avant-garde, complete, different, disruptive, entire, factious, far-reaching, fresh, futuristic, groundbreaking, imaginative, innovational, innovatory, inventive, modern, new, original, pioneering, profound, seditious, state-of-the-art, subversive, sweeping, trailblazing, unconventional, unorthodox, unusual, wide-ranging
rich: abundant, arable, copious, fertile, full, generous, plentiful, profitable, profit-making, profuse, propertied, prosperous, vivid
rigorous: attentive, austere, bad, bleak, conscientious, correct, cruel, demanding, despotic, diligent, exact, exacting, extreme, harsh, mathematical, methodical, particular, perfectionist, precise, relentless, rigid, scrupulous, stringent, ultra-careful, uncompromising, unsparing
robust: long-lasting, resilient, vigorous
safer: attentive, benign, cautious, defended, harmless, impregnable, innocuous, invulnerable, low-risk, mild, non-irritant, non-poisonous, non-toxic, prudent, risk-free, riskless, sheltered, shielded, unassailable, undamaged, unharmed, unhurt, uninjured, unscathed
scientific: controlled, exact, mathematical, methodical, ordered, orderly, organized, precise, regulated, technical, technological
senior: chief, elder, higher-ranking, highest-ranking, high-status, older, superior
significant: consequential, expressive, indicative, informative, knowing, noteworthy, pithy, purposeful, revealing, valid
skilled: able, accomplished, adept, adroit, capable, competent, deft, dexterous, expert, gifted, practised, proficient, smart, trained, versed
sophisticated: innovatory, trailblazing, worldly

© 2024 Qiu HS et al. *JAMA Network Open.*

stark: arid, austere, bare, barren, bleak, blunt, complete, crisp, desolate, distinct, empty, evident, grim, harsh, obvious, outright, plain, positive, pure, sharp, sheer, simple, sombre, striking, unadorned, uncomfortable, undecorated, unembellished, unvarnished, vacant, vigorous
stellar: singular, staggering, stunning
strategic: calculated, deliberate, planned
strong: able, accomplished, adept, adroit, biting, capable, cogent, concentrated, deep-seated, drastic, dynamic, eager, earnest, extreme, formidable, full, glaring, heady, healthy, hearty, impenetrable, impregnable, indestructible, inviolable, loud, plausible, potent, proficient, profound, pungent, redoubtable, resolute, sharp, solid, staunch, steadfast, stiff, striking, tenacious, thriving, unassailable, undiluted, valid, vehement, vigorous, vivid, zealous
substantial: appreciable, decent, long-lasting, marked, profitable, profit-making, prosperous, real, sizeable, solid, useful, valuable, worthwhile
successful: burgeoning, flourishing, gainful, lucrative, moneymaking, profitable, profit-making, prosperous, solvent, thriving, triumphant, victorious
surprising: agape, confounding, jolted, jolting, nonplus, shocked, shocking, stagger, staggered, stunning, unanticipated, unexpected, unforeseen, unpredicted
sustainable: acceptable, dependable, just, justified, legitimate, reasonable, reliable, sensible, sure, trustworthy, valid
synergy: accord, association, collaboration, compromise, concord, concurrence, coopetition, coordination, liaison, partnership, teamwork, understanding, unity
systematic: coherent, consistent, fastidious, formal, logical, methodical, orderly, organized, planned, practical, regular, routine, standard, standardized, structured, systematized
tailored: adapted, adjusted, altered, attuned, changed, converted, fitted, geared, modified, moulded, reshaped, shaped, suited, tuned
talented: able, accomplished, adept, adroit, apt, capable, competent, consummate, deft, dexterous, expert, gifted, polished, proficient
tangible: appreciable, concrete, corporeal, definite, discernible, distinct, evident, indisputable, intelligible, manifest, measurable, obvious, palpable, perceptible, physical, positive, real, solid, striking, tactile, touchable, undoubted, unmistakable, verifiable
timely: appropriate, apt, convenient, expedient, felicitous, fitting, opportune, prompt, punctual, suitable
top: best, chief, choice, commanding, elite, excellent, foremost, high, highest, high-grade, leading, main, maximal, maximum, peerless, pre-eminent, prime, principal, quality, select, superior, superlative, top-class, top-grade, topmost, top-quality, top-tier, unexcelled, unsurpassed, upmost, upper, uppermost, utmost
transformative: alternative, changable, converted, metamorphosing, modifiable, mutatative, transfigurative, transmutative
tremendous: excellent, prodigious
ultimate: best, central, concluding, conclusive, definitive, elemental, end, endmost, eventual, furthest, highest, last, optimum, primary, prime, quintessential, superlative, supreme, terminal, topmost, unrivalled, unsurpassed, utmost
unanswered: disputed, pending, undecided, undetermined, unresolved, unsettled
unique: distinctive, eccentric, idiosyncratic, individual, isolated, noteworthy, particular, peculiar, quirky, signal, singular, special, specific
unparalleled: rare, singular, unequalled
unprecedented: unequalled, unmatched, unrivalled
urgent: dogged, drastic, emergency, extreme, grave, high-priority, obstinate, persistent, pressing, resolute, tenacious, top-priority, unrelenting
user-friendly: articulate, coherent, comprehensible, crystalline, intelligible, lucid, understandable
vast: immeasurable, limitless, prodigious, wide
vibrant: animated, blaring, dynamic, echoing, effervescent, full, pulsating, quaking, quavering, quavery, quivering, reverberant, reverberating, stimulating, strident, striking, vigorous, vivid
vital: active, animated, consequential, dynamic, high-priority, life-preserving, life-sustaining, mandatory, necessary, needed, pre-eminent, pressing, required, requisite, vigorous

© 2024 Qiu HS et al. *JAMA Network Open.*

eTable 3. Logistic Regression of the Odds of Being Funded (NNF Grants)

	Full Control Model		Partial Control Model		Bivariate Model		
% Promotional Words	1.464	***	1.369	***	1.326	***	*Coef*
	(0.118)		(0.101)		(0.085)		*SE*
	[1.250- 1.714]		[1.186 - 1.582]		[1.169 - 1.504]		*CI*
	0.00		0.00		0.00		*p-value*
Gender (M=1)	0.909		0.891				
	(0.061)		(0.059)				
	[0.797- 1.037]		[0.782 - 1.015]				
	0.16		0.08				
	(0.105)		(0.103)				
	[0.914 - 1.327]		[0.907 - 1.312]				
	0.31		0.36				
# Publications (lrg)	1.046		0.998				
	(0.123)		(0.117)				
	[0.830 - 1.318]		[0.794 - 1.255]				
	0.70		0.99				
# Citations (v.sml)	0.850		0.830				
	(0.098)		(0.095)				
	[0.677 - 1.065]		[0.663 - 1.039]				
	0.16		0.10				
# Citations (sml)	1.365	**	1.355	**			
	(0.166)		(0.163)				
	[1.076 - 1.732]		[1.071 - 1.715]				
	0.01		0.01				
# Citations (med)	1.646	***	1.654	***			
	(0.217)		(0.216)				
	[1.271 - 2.131]		[1.281 - 2.136]				
	0.00		0.00				
# Citations (lrg)	2.062	***	2.082	***			
	(0.305)		(0.305)				
	[1.543 - 2.754]		[1.563 - 2.774]				
	0.00		0.00				
# Prior Applications (sml)	0.908		0.914				
	(0.085)		(0.084)				
	[0.757 - 1.090]		[0.762 - 1.095]				
	0.30		0.33				
# Prior Applications (lrg)	0.823	**	0.815	**			
	(0.078)		(0.077)				
	[0.684 - 0.990]		[0.678 - 0.980]				
	0.04		0.03				
# Grants Funded (sml)	2.124	***	2.128	***			
	(0.204)		(0.203)				
	[1.759 - 2.564]		[1.764 - 2.566]				
	0.00		0.00				
# Grants Funded (lrg)	2.545	***	2.518	***			
	(0.308)		(0.303)				
	[2.007 - 3.227]		[1.989 - 3.188]				
	0.00		0.00				
Novelty Score	1.002		1.003				

© 2024 Qiu HS et al. *JAMA Network Open.*

	(0.003)		(0.003)			
	[0.997 - 1.007]		[0.997 - 1.008]			
	0.45		0.31			
Applied For Amt (v.sml)	0.683	***	0.808	**		
	(0.076)		(0.084)			
	[0.549 - 0.849]		[0.659 - 0.991]			
	0.00		0.04			
Applied For Amt (sml)	0.577	***	0.726	***		
	(0.067)		(0.077)			
	[0.459 - 0.724]		[0.590 - 0.894]			
	0.00		0.00			
Applied For Amt (med)	0.742	**	0.993			
	(0.087)		(0.103)			
	[0.589 - 0.934]		[0.810 - 1.218]			
	0.01		0.95			
Applied For Amt (lrg)	0.273	***	0.492	***		
	(0.041)		(0.055)			
	[0.204 - 0.365]		[0.396 - 0.612]			
	0.00		0.00			
Flesch Reading Score	0.986					
	(0.028)					
	[0.932 - 1.043]					
	0.62					
Concreteness Score	0.956					
	(0.028)					
	[0.903 - 1.011]					
	0.12					
# Words Fixed Effects	Yes					
Year Fixed Effects	Yes		Yes		Yes	
Intercept	0.157	***	0.204	***	0.200	***
	(0.024)		(0.026)		(0.020)	
	[0.117 - 0.211]		[0.159 - 0.264]		[0.164 - 0.243]	
	0.00		0.00		0.00	
Number of observations	7752		7752		9096	

*** p<.001, * p<.01

© 2024 Qiu HS et al. *JAMA Network Open.*

eTable 4. Logistic Regression of the Odds of Being Funded (NIH Grants)

	Full Control Model		Bivariate Model	
% Promotional Words	1.518	*	1.427	*
	(0.253)		(0.211)	
	[1.095 - 2.105]		[1.068 - 1.905]	
	0.01		0.02	
Gender (M=1)	0.993			
	(0.121)			
	[0.782 - 1.260]			
	0.95			
# Publications (sml)	0.944			
	(0.296)			
	[0.511 - 1.745]			
	0.85			
# Publications (lrg)	1.062			
	(0.369)			
	[0.538 - 2.098]			
	0.86			
# Citations (v.sml)	0.788			
	(0.249)			
	[0.424 - 1.465]			
	0.45			
# Citations (sml)	1.189			
	(0.423)			
	[0.592 - 2.387]			
	0.63			
# Citations (med)	1.208			
	(0.450)			
	[0.582 - 2.507]			
	0.61			
# Citations (lrg)	1.431			
	(0.541)			
	[0.682 - 3.003]			
	0.34			
Amt Applied For (v.sml)	0.726			
	(0.122)			
	[0.522 - 1.009]			
	0.06			
Amt Applied For (sml)	0.491	**		
	(0.091)			
	[0.343 - 0.705]			
	0.00			
Amt Applied For (med)	0.635	*		
	(0.114)			
	[0.447 - 0.902]			
	0.01			
Amt Applied For (lrg)	1.038			
	(0.347)			
	[0.539 - 1.999]			
	0.91			
Flesch Reading_Score	0.713	**		
	(0.057)			
	[0.609 - 0.834]			
	0.00			

© 2024 Qiu HS et al. *JAMA Network Open.*

Concreteness Score	0.946	
	(0.057)	
	[0.840 - 1.065]	
	0.36	
# Words Fixed Effects	Yes	
Year Fixed Effects	Yes	Yes
	[0.141 - 0.930]	[0.138 - 0.357]
	0.03	0.00
Number of observations	2250	2438

** p<.01, * p<.05

© 2024 Qiu HS et al. *JAMA Network Open.*

eTable 5. Predictors of the Percentage of Promotional Words in a Grant (NIH)

	NNF		NIH	
% Promotional Words in Grant				
Gender (M=1)	0.045	**	0.041	**
	(0.009)		(0.015)	
	[0.026 - 0.063]		[0.012 - 0.069]	
	0.00		0.00	
Age 30-39	-0.112	*		
	(0.049)			
	[-0.208 - 0.015]			
	0.02			
Age 40-49	-0.201	**		
	(0.049)			
	[-0.297 - -0.105]			
	0.00			
Age 50-59	-0.234	**		
	(0.049)			
	[-0.331 - -0.138]			
	0.00			
Age 60-69	-0.270	**		
	(0.050)			
	[-0.369 - -0.172]			
	0.00			
Age >69	-0.359	**		
	(0.061)			
	[-0.478 - -0.239]			
	0.00			
# Publications (sml)	0.007		-0.060	
	(0.013)		(0.034)	
	[-0.017 - 0.032]		[-0.127 - 0.007]	
	0.56		0.08	
# Publications (lrg)	-0.017		-0.045	
	(0.017)		(0.039)	
	[-0.050 - 0.015]		[-0.121 - 0.031]	
	0.30		0.24	
# Citations (v.sml)	-0.025		-0.051	
	(0.015)		(0.035)	
	[-0.05 - 0.004]		[-0.120 - 0.018]	
	0.09		0.14	
# Citations (sml)	-0.014		-0.056	
	(0.016)		(0.039)	
	[-0.047 - 0.018]		[-0.133 - 0.021]	
	0.39		0.15	
# Citations (med)	0.030		-0.070	
	(0.018)		(0.041)	
	[-0.006 - 0.066]		[-0.151 - 0.011]	
	0.11		0.09	
# Citations (lrg)	0.067	**	-0.063	
	(0.021)		(0.042)	
	[0.025 - 0.108]		[-0.146 - 0.020]	
	0.00		0.14	
# Prior Apps (sml)	-0.010			
	(0.012)			
	[-0.035 - 0.014]			

© 2024 Qiu HS et al. *JAMA Network Open.*

	0.42			
# Prior Apps (lrg)	-0.027	*		
	(0.012)			
	[-0.051 - -0.002]			
	0.03			
# Prior Grants Funded (sml)	0.052	**		
	(0.014)			
	[0.023 - 0.080]			
	0.00			
# Prior Grants Funded (lrg)	0.011			
	(0.019)			
	[-0.026 - 0.049]			
	0.55			
Amt Applied For (v.sml)	0.052	**	-0.019	
	(0.016)		(0.021)	
	[0.021 - 0.084]		[-0.059 - 0.021]	
	0.00		0.36	
Amt Applied For (sml)	0.075	**	-0.064	**
	(0.016)		(0.021)	
	[0.043 - 0.108]		[-0.106 - -0.022]	
	0.00		0.00	
Amt Applied For (med)	0.081	**	-0.033	
	(0.017)		(0.022)	
	[0.047 - 0.114]		[-0.075 - 0.010]	
	0.00		0.14	
Amt Applied For (lrg)	0.205	**	0.078	
	(0.020)		(0.042)	
	[0.166 - 0.244]		[-0.004 - 0.160]	
	0.00		0.06	
Novelty Score	0.017	**		
	(0.006)			
	[0.006 - 0.029]			
	0.00			
Flesch Reading Score	-0.022	**	0.034	**
	(0.005)		(0.010)	
	[-0.03 - -0.013]		[0.014 - 0.053]	
	0.00		0.00	
Concreteness Score	0.126	**	0.079	**
	(0.004)		(0.007)	
	[0.119 - 0.134]		[0.065 - 0.093]	
	0.00		0.00	
# Words Fixed Effects	Yes		Yes	
Year Fixed Effects	Yes		Yes	
Intercept	1.022	**	1.087	**
	(0.051)		(0.057)	
	[0.923 - 1.121]		[0.975 - 1.199]	
	0.00		0.00	
Number of observations	7752		2252	
R-squared	0.23		0.14	

** p<.01, * p<.05

© 2024 Qiu HS et al. *JAMA Network Open.*

eReferences.

1. Oliveira DF, Ma Y, Woodruff TK, Uzzi B. Comparison of National Institutes of Health grant amounts to first-time male and female principal investigators. *Jama*. 2019;321(9):898-900.
2. Wu L, Wang D, Evans JA. Large teams develop and small teams disrupt science and technology. *Nature*. 2019;566(7744):378.
3. Yang Y, Tian TY, Woodruff TK, Jones BF, Uzzi B. Gender-diverse teams produce more novel and higher-impact scientific ideas. *Proceedings of the National Academy of Sciences*. 2022;119(36):e2200841119.
4. Uzzi B, Mukherjee S, Stringer M, Jones B. Atypical combinations and scientific impact. *Science*. 2013;342(6157):468-472.
5. Kim D, Cerigo DB, Jeong H, Youn H. Technological novelty profile and invention's future impact. *EPJ Data Science*. 2016;5(1):1-15.
6. Peng H, Qiu HS, Fosse HB, Uzzi B. Promotional language and the adoption of innovative ideas in science. *Proceedings of the National Academy of Sciences*. 2024;121(25):e2320066121.
7. Wang J, Veugelers R, Stephan P. Bias against novelty in science: A cautionary tale for users of bibliometric indicators. *Research Policy*. 2017;46(8):1416-1436.
8. Brysbaert M, Warriner AB, Kuperman V. Concreteness ratings for 40 thousand generally known English word lemmas. *Behavior research methods*. 2014;46:904-911.
9. Millar N, Salager-Meyer F, Budgell B. "It is important to reinforce the importance of…":'Hype'in reports of randomized controlled trials. *English for Specific Purposes*. 2019;54:139-151.
10. Millar N, Batalo B, Budgell B. Trends in the use of promotional language (hype) in abstracts of successful national institutes of health grant applications, 1985-2020. *JAMA Network Open*. 2022;5(8):e2228676-e2228676.

© 2024 Qiu HS et al. *JAMA Network Open.*
