# Supplementary material for: Use of Promotional Language in Grant Applications and Grant Success
Source: JAMA Netw Open. 2024 Dec 11;7(12):e2448696. doi: 10.1001/jamanetworkopen.2024.48696 (PMC11635532; doi:10.1001/jamanetworkopen.2024.48696)
Supplement: Supplement 2. — Data Sharing Statement [file jamanetwopen-e2448696-s002.pdf]

Data Sharing Statement

Qiu. Use of Promotional Language in Grant Applications and Grant Success. *JAMA Netw Open*. Published December 11, 2024. doi:10.1001/jamanetworkopen.2024.48696

Data
Data available: Yes
Data types: Deidentified participant data
How to access data: Please email Dr. Brian Uzzi uzzi@kellogg.northwestern.edu for data.
When available: With publication

Supporting Documents
Document types: Statistical/analytic code
How to access documents: We will publish the data on GitHub when the paper is accepted.
When available: With publication

Additional Information
Who can access the data: Researchers requesting the data.
Types of analyses: Scientific research.
Mechanisms of data availability: After a request has been made.
